# Polarization-controlled tunable directional spin-driven photocurrents in a magnetic metamaterial with threefold rotational symmetry

**DOI:** 10.1038/s41467-022-34374-7

**Published:** 2022-11-07

**Authors:** Masakazu Matsubara, Takatsugu Kobayashi, Hikaru Watanabe, Youichi Yanase, Satoshi Iwata, Takeshi Kato

**Affiliations:** 1 Department of Physics, Tohoku University, Sendai, 980-8578 Japan; 2 Center for Science and Innovation in Spintronics, Tohoku University, Sendai, 980-8577 Japan; 3 Research Center for Advanced Science and Technology, University of Tokyo, Tokyo, 153-8904 Japan; 4 Department of Physics, Graduate School of Science, Kyoto University, Kyoto, 606-8502 Japan; 5 Institute for Molecular Science, Okazaki, 444-8585 Japan; 6 Institute of Materials and Systems for Sustainability, Nagoya University, Furo-cho, Chikusa-ku, Nagoya, 464-8603 Japan

**Keywords:** Spintronics, Metamaterials, Magnetic properties and materials

## Abstract

Future spintronics and quantum technologies will require a portfolio of techniques for manipulating electron spins in functional nanodevices. Especially, the establishment of the methods to control spin current is the key ingredient essential for the transfer and processing of information, enabling faster and low-energy operation. However, a universal method for manipulating spin currents with full-directional controllability and tunable magnitude has not been established. Here we show that an artificial material called a magnetic metamaterial (MM), which possesses a novel spintronic functionality not exhibited by the original substance, generates photo-driven ultrafast spin currents at room temperature via the magneto-photogalvanic effect. By tuning the polarization state of the excitation light, these spin currents can be directed with tunable magnitude along an arbitrary direction in the two-dimensional plane of the MM. This new concept may guide the design and creation of artificially engineered opto-spintronic functionalities beyond the limitations of conventional material science.

## Abstract (web summary)

By carefully structuring and patterning a material, it is possible to introduce emergent properties that would otherwise not exist. These metamaterials have allowed the development of a wide variety of new optical properties. Here, Matsubara et al present a magnetic metamaterial, where spin-currents can be directed by tuning the polarization of the incident light.

## Introduction

Manipulation of electron spin polarization is an essential topic of spintronics^1–3^. An attractive approach to generating and controlling spin currents is the use of light, and new principles of light conversion into spin currents have been explored, including a light-helicity-dependent control of spin-polarized currents in semiconductors^4^ and topological insulators^5,6^, laser-generated superdiffusive spin currents in magnetic multilayers^7^, propagation control of spin waves in a magnetic insulator^8^, generation of pure spin currents by optical quantum interference effect^9^ and surface plasmon excitation^10^, and spin-dependent scattering^11,12^. In the last of these techniques, pure spin currents are converted into spin-polarized DC electric currents under an applied magnetic field, generating the magneto-photogalvanic effect (MPGE)^13–15^. However, a universal method for manipulating spin currents with full-directional controllability and tunable magnitude, independent of specific material properties and not limited to low-temperature operation, has not been established.

Advances in nanofabrication technology can overcome the fundamental limitations imposed by nature. Metamaterials are engineered artificial materials that elicit new responses, not exhibited by the original substance, using tailored subwavelength structures^16^. The required material functionality can be achieved by designing the shape, size, period, and mutual arrangement of the constituent nanostructures. This deceptively simple yet powerful concept enables the realization of many unprecedented optical properties such as negative refractive index^17^, super-resolution imaging^18^, cloaking^19^ and perfect absorption of electromagnetic waves^20^.

During the past 20 years, metamaterials have entered the realm of nonlinear optics^21^. Especially, the introduction of noncentrosymmetry enables second-order nonlinear optical effects such as second harmonic generation (SHG), which requires, in the leading order, the breaking of space inversion symmetry. Consequently, nonlinear optical processes forbidden in centrosymmetric media are allowable in metamaterials^22^. The introduction of magnetism into noncentrosymmetric metamaterials causes attractive magneto-optical responses such as magnetic-field controllable SHG^23,24^ and nonreciprocal optical magnetoelectric effect^25^, originating from the breaking of both space inversion and time-reversal symmetries. The MPGE, which are used in this study as the principle of photo-driven ultrafast spin current generation, can be allowed under the same conditions. The MPGE (called the magnetic ratchet effect in artificial periodic structures) has been detected in metamaterials with nonmagnetic superlattices^26,27^. However, it has never been observed in magnetic metamaterials (MMs).

In this study, we report the observation of MPGE in noncentrosymmetric MMs with artificially built-in threefold rotational symmetry and out-of-plane magnetization. Reflecting this particular symmetry and remanent zero-field magnetization, the direction and magnitude of spin-driven and potentially spin-polarized photocurrents are fully controlled without any external bias fields by tuning the polarization state of the excitation light. The spin-switchable nature of photocurrents under weak external magnetic fields allows for the additional functionality of spin current manipulation. Our experimental results, which are entirely consistent with symmetry predictions, show that nanoscale symmetry engineering can convert already known magnets into functional opto-spintronic materials working at room temperature.

## Results

### Magneto-photogalvanic effect and magnetic metamaterial design

The MPGE is a second-order nonlinear optical effect and can be phenomenologically expressed as^13–15^

1 $${J}_{i}={\beta }_{ijk}^{{{{{{{{{{{{\rm{m}}}}}}}}}}}}}(0;\omega,-\omega ){E}_{j}(\omega ){E}_{k}^{*}(\omega ).$$

Here, *E*_*j*_(*ω*) and ${E}_{k}^{*}(\omega )$ = *E*_*k*_( − *ω*) denote the *j*- and *k*-polarized electric fields, respectively, of the incident light wave at *ω*. These fields induce the *i*-directed zero-bias photocurrent *J*_*i*_ proportional to the square of the AC electric field; that is, to the intensity of the light. The third-rank polar tensor ${\beta }_{ijk}^{{{{{{{{{{{{\rm{m}}}}}}}}}}}}}$ changes the sign under either a time-reversal or space-inversion operation, and is determined by the magneto-crystalline symmetry of the system. Unlike the conventional (nonmagnetic) photogalvanic effect, in which the photocurrent direction reverses only under a space-inversion operation, the photocurrent driven by the MPGE reverses under both space-inversion and time-reversal operations. This makes the MPGE photocurrent in magnetic materials both spin-driven and spin-switchable^15^. A flexible nanostructure design not only elicits the second-order nonlinear optical effects in metamaterials^28^, but also enables the manipulation of photo-driven ultrafast spin currents via the MPGE in properly designed MMs. This sophisticated manipulation is possible because the carriers in ferro- and ferri-magnetic materials carry a net spin angular momentum under an external DC electric field.

**Fig. 1:**
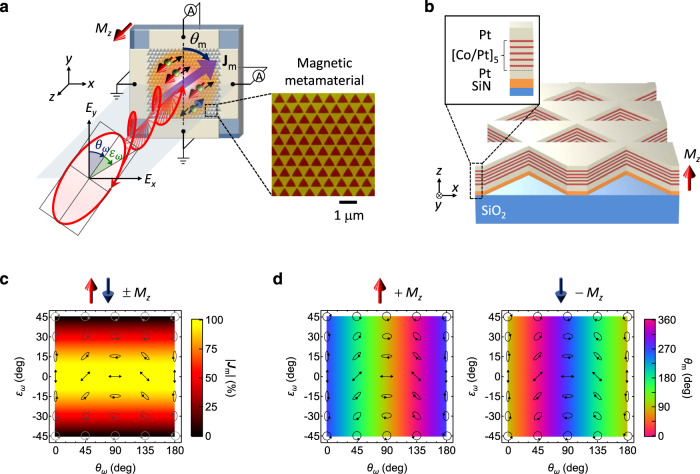
Polarization-controlled tunable directional spin-driven photocurrents in a magnetic metamaterial. **a** Schematic of the experimental setup for generating spin-driven photocurrents. A normally incident light with an arbitrarily polarized state (polarization angle *θ*_*ω*_, ellipticity angle *ε*_*ω*_) excites a magnetic metamaterial (MM) with threefold rotational symmetry and out-of-plane magnetization *M*_*z*_, generating spin-driven photocurrents **J**_m_. The flow direction *θ*_m_ and magnitude ∣ **J**_m_∣ of **J**_m_ can be independently controlled by *θ*_*ω*_ and *ε*_*ω*_, respectively, via the magneto-photogalvanic effect. The inset shows an atomic force microscope image of the MM. **b** Schematic of the MM with a centrosymmetric Co/Pt ferromagnetic metallic multilayer film, showing the direction of *M*_*z*_. The MM consists of arrayed nanostructures (antidot lattice) of triangular holes with side lengths of 480 nm. The period of the triangular lattice is 558 nm. **c**, **d** 2D color maps of (**c**) ∣ **J**_m_∣ and (**d**) *θ*_m_ on the *θ*_*ω*_ vs *ε*_*ω*_ plane. The polarization states *θ*_*ω*_ and *ε*_*ω*_ of the excitation light are shown as black overlays. ∣ **J**_m_∣ can be modulated by *ε*_*ω*_ ($|{{{{{{{{{{{{\bf{\,J}}}}}}}}}}}}}_{{{{{{{{{{{{\rm{m}}}}}}}}}}}}}|\propto \cos 2{\varepsilon }_{\omega }$) for ± *M*_*z*_. Meanwhile, when *β*^m^ > 0, *θ*_m_ can be written as *θ*_m_ = − 2*θ*_*ω*_ − 90° for + *M*_*z*_ (left panel) and *θ*_m_ = − 2*θ*_*ω*_ + 90° for − *M*_*z*_ (right panel), leading to full-directional control by *θ*_*ω*_. Reversal of *M*_*z*_ does not change ∣ **J**_m_∣ but flips *θ*_m_, indicating direct coupling between the spin polarization direction and *θ*_m_.

To demonstrate this novel artificially engineered opto-spintronic functionality, we designed a model MM system that generates polarization-controlled tunable directional spin-driven photocurrents (Fig. 1a). We fabricated the MM with a centrosymmetric Co/Pt ferromagnetic metallic multilayer film with out-of-plane magnetization (Fig. 1b). It consists of periodic triangle-hole-arrayed nanostructures (antidot lattice) with threefold rotational symmetry. This particular symmetry is necessary to control the direction and magnitude of zero-bias photocurrents due to angular momentum conservation in second-order nonlinear optical processes^28^. The out-of-plane magnetization preserves the artificially built-in threefold rotational symmetry of the MM. Owing to the exchange splitting of the conduction band, carriers in the Co/Pt multilayer film are spin-polarized with an approximate polarization degree of 50%^29^ (the ratio of spin-up and spin-down electron densities near the Fermi level is ~ 3: 1).

When an arbitrarily polarized incident light (polarization angle *θ*_*ω*_, ellipticity angle *ε*_*ω*_) excites a MM with threefold rotational symmetry and out-of-plane magnetization *M*_*z*_, zero-bias spin-driven photocurrents **J**_m_ can be generated via the MPGE (Fig. 1a). Considering the magnetic point group of the MM as $3m^{\prime}$ and assuming only one independent MPGE tensor component *β*^m^, the magnitude ∣ **J**_m_∣ and flow direction *θ*_m_ of **J**_m_ under ± *M*_*z*_ are respectively defined as^30^

2 $$|{{{{{{{{{{{{\bf{\,J}}}}}}}}}}}}}_{{{{{{{{{{{{\rm{m}}}}}}}}}}}}} |=|{\beta }^{{{{{{{{{{{{\rm{m}}}}}}}}}}}}}|{{{{{{{{{\rm{cos}}}}}}}}}} 2{\varepsilon }_{\omega }\qquad (-4{5}^{\circ }\le {\varepsilon }_{\omega }\le 4{5}^{\circ }),$$

3 $${\theta }_{{{{{{{{{{{{\rm{m}}}}}}}}}}}}}=\left\{\begin{array}{ll}-2{\theta }_{\omega }\mp 9{0}^{\circ }\qquad \quad &({\beta }^{{{{{{{{{{{{\rm{m}}}}}}}}}}}}} > 0)\\ -2{\theta }_{\omega }\pm 9{0}^{\circ }\qquad \quad &({\beta }^{{{{{{{{{{{{\rm{m}}}}}}}}}}}}} < 0)\end{array}\right.,$$

where ∣ **J**_m_∣ and *θ*_m_ can be independently controlled by *ε*_*ω*_ and *θ*_*ω*_ of the excitation light, respectively (see Methods for a symmetry analysis). Figure 1c, d plot ∣ **J**_m_∣ and *θ*_m_ as functions of the polarization state of the excitation light, respectively, when *β*^m^ > 0. Note that reversing *M*_*z*_ does not change ∣ **J**_m_∣ but flips *θ*_m_. Therefore, the direction and magnitude of the spin-driven photocurrents can be controlled and tuned, respectively, by controlling the polarization state of the excitation light. These relations are purely determined by the artificially built-in symmetry of the MM and (unlike the valley-dependent optical selection rules in transition metal dichalcogenides^31^) are not restricted to specific material properties or excitation wavelength.

### Zero-bias spin-driven photocurrents

**Fig. 2:**
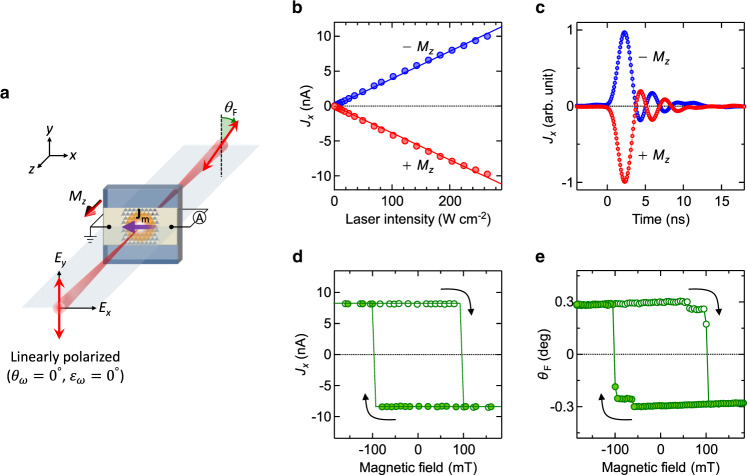
Fundamental properties of spin-driven photocurrents. **a** Schematic of the experimental setup for measuring zero-bias photocurrent *J*_*x*_ and Faraday rotation *θ*_F_ at normal incidence of linearly *y*-polarized (*θ*_*ω*_ = *ε*_*ω*_ = 0°) 800 nm light. **b** Laser-intensity dependences of *J*_*x*_ for opposite out-of-plane saturation magnetizations ± *M*_*z*_. The lines are linearly fitted to the data. **c** Dynamical responses of *J*_*x*_ for ± *M*_*z*_. The response time is limited to the bandwidth of the instruments used. **d**, **e** Out-of-plane magnetic-field dependences of (**d**) *J*_*x*_ and (**e**) *θ*_F_. The two-step behaviors in *θ*_F_ arise from the experimental conditions: the incident laser beam hits not only the MM but also slightly the surrounding unstructured Co/Pt multilayer film with a smaller magnetic coercive field than that of the MM (see Supplementary Fig. 2). Two-step behavior is not observed in *J*_*x*_ in (**d**) because the unstructured Co/Pt multilayer film has a centrosymmetric structure and therefore does not contribute to the zero-bias photocurrent, which requires the breaking of space inversion symmetry.

The MM was illuminated by a normal incident 800-nm femtosecond laser pulse (see Methods for experimental details). First, we measured the photocurrent *J*_*x*_ along the *x* direction across the unbiased MM with opposite out-of-plane saturation magnetization ± *M*_*z*_ under linearly *y*-polarized irradiation (*θ*_*ω*_ = *ε*_*ω*_ = 0°) (Fig. 2a). The magnitude of the detected zero-bias photocurrent scales linearly with light intensity (Fig. 2b). The light-to-current conversion ratio ( ~ 40 pA W^−1^cm^2^) is similar to that obtained by the circular photogalvanic effect in Bi_2_Se_3_^5^, where spin-polarized photocurrents were induced by optically driving the Dirac cone with oblique-incidence circularly polarized light. In the present experiments, all helicity-dependent spin photocurrents, such as the spin-galvanic effect^4^ and circular photogalvanic effect^5^ are forbidden because the light is linearly polarized. In addition, the photocurrent observed here cannot be explained by the conventional (nonmagnetic) photogalvanic effect^13^ (see Supplementary Figs. 3, 4, 6 and Supplementary Discussion) or by the shift current mechanism^32,33^. The photocurrent, whose direction is flipped by the time-reversal operation (reversal of *M*_*z*_, Fig. 2b), can be explained only by the MPGE. We confirmed that the nonstructured centrosymmetric Co/Pt multilayer film do not generate zero-bias photocurrents for excitation light of any polarization.

The artificially built-in asymmetry in our MM imbalances the distribution of photoexcited carriers between the states with positive and negative wavevectors in the spin-up and spin-down subbands, causing electrons flows within each spin subband. As spin-up and spin-down electrons move in opposite directions through spin-dependent scattering, spin currents are generated^11,12^. In a spin-polarized system, different populations of the two spin subbands yield a net electron flow. Thus, spin polarization of the zero-bias photocurrents can be detected as an electric current whose direction is switched by reversing *M*_*z*_. Time-resolved measurements reveal a sub-nanosecond response, although the rise- and fall-time responses are limited by the bandwidth of the instruments used in this experiment (Fig. 2c).

To clarify the coupling of the photocurrent to *M*_*z*_, the magnetic-field dependence of *J*_*x*_ is plotted in Fig. 2d. The magnitude and sign of *J*_*x*_ suddenly change while sweeping the external out-of-plane magnetic field, yielding a ferromagnetic hysteresis with an antisymmetric closed loop. This behavior directly reflects spin polarization in the conduction band of the MM, as further evidenced by the measured magnetic-field dependence of the Faraday rotation *θ*_F_ (Fig. 2e). Therefore, the flow direction of the zero-bias photocurrents generated in our MM is directly coupled to the spin-polarization direction.

### Full-directional control of the spin-driven photocurrents

**Fig. 3:**
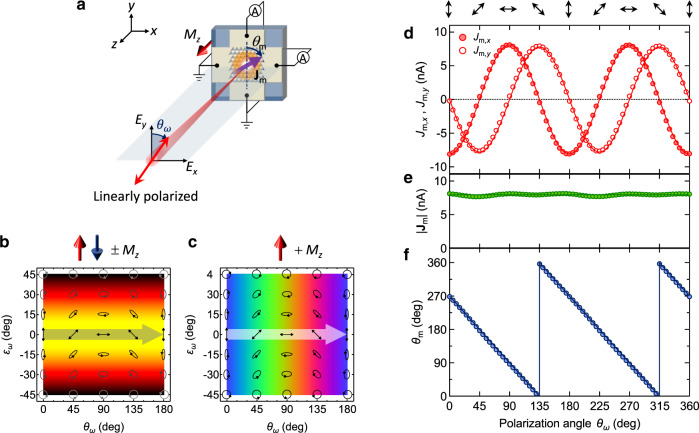
Full-directional control of spin-driven photocurrents. **a** Schematic of the experimental setup for measuring zero-bias photocurrents along the *x* and *y* directions at normal incidence of continuously rotated linearly polarized 800-nm light. **b**, **c** 2D color maps of (**b**) ∣ **J**_m_∣ for ± *M*_*z*_ and (**c**) *θ*_m_ for + *M*_*z*_ when linearly polarized light crosses the horizontal directions (marked by thick arrows). **d** *θ*_*ω*_ dependence of *J*_m,*x*_ and *J*_m,*y*_, calculated as ${J}_{{{{{{{{{{{{\rm{m}}}}}}}}}}}},x}=\left[{J}_{x}(+{M}_{z})-{J}_{x}(-{M}_{z})\right]/2$ and ${J}_{{{{{{{{{{{{\rm{m}}}}}}}}}}}},y}=\left[{J}_{y}(+{M}_{z})-{J}_{y}(-{M}_{z})\right]/2$, respectively, to exclude conventional (nonmagnetic) photogalvanic effects (see Supplementary Figs. 3, 4, 6 and Supplementary Discussion). The lines are fitted to ${J}_{{{{{{{{{{{{\rm{m}}}}}}}}}}}},x}\propto -\cos 2{\theta }_{\omega }$ and ${J}_{{{{{{{{{{{{\rm{m}}}}}}}}}}}},y}\propto -\sin 2{\theta }_{\omega }$. **e** ∣ **J**_m_∣ calculated as $|{{{{{{{{{{{{\bf{\,J}}}}}}}}}}}}}_{{{{{{{{{{{{\rm{m}}}}}}}}}}}}} |=\sqrt{{(\,{J}_{{{{{{{{{{{{\rm{m}}}}}}}}}}}},x})}^{2}+{(\,{J}_{{{{{{{{{{{{\rm{m}}}}}}}}}}}},y})}^{2}}$ and $|{{{{{{{{{{{{\bf{\,J}}}}}}}}}}}}}_{{{{{{{{{{{{\rm{m}}}}}}}}}}}}} |={{{{{{{{{{{\rm{const.}}}}}}}}}}}}$ **f** *θ*_m_ calculated as ${\theta }_{{{{{{{{{{{{\rm{m}}}}}}}}}}}}}={\tan }^{-1}\left(\,{J}_{{{{{{{{{{{{\rm{m}}}}}}}}}}}},x}/{J}_{{{{{{{{{{{{\rm{m}}}}}}}}}}}},y}\right)$ (blue circles) and fitted to *θ*_m_ = − 2*θ*_*ω*_ − 90° (blue lines).

To demonstrate the directional controllability of the spin-driven photocurrents, we rotated the polarization plane of the linearly polarized light (*θ*_*ω*_ = 0° − 360°, *ε*_*ω*_ = 0°) and separately detected the photocurrents along the *x* and *y* directions (Fig. 3a). According to Eqs. (2) and (3), continuous rotation of *θ*_*ω*_ through 180° achieves full-directional control of **J**_m_ with constant magnitude (Fig. 3b and c). When plotted against *θ*_*ω*_, the spin-driven photocurrents *J*_m,*x*_ and *J*_m,*y*_ exhibits $-{{{{{{{{{\rm{cos}}}}}}}}}}\, 2{\theta }_{\omega }$ and $-{{{{{{{{{\rm{sin}}}}}}}}}}\,2{\theta }_{\omega }$ behaviors, respectively (Fig. 3d). Reflecting the nearly identical amplitudes of *J*_m,*x*_ and *J*_m,*y*_, ∣ **J**_m_∣$(=\sqrt{{(\,{J}_{{{{{{{{{{{{\rm{m}}}}}}}}}}}},x})}^{2}+{(\,{J}_{{{{{{{{{{{{\rm{m}}}}}}}}}}}},y})}^{2}})$ is almost independent of *θ*_*ω*_ (Fig. 3e). In contrast, *θ*_m_$(={{{{{{{{{{\rm{tan}}}}}}}}}} }^{-1}(\,{J}_{{{{{{{{{{{{\rm{m}}}}}}}}}}}},x}/{J}_{{{{{{{{{{{{\rm{m}}}}}}}}}}}},y}))$ is a continuous function of *θ*_*ω*_; specifically, *θ*_m_ = − 2*θ*_*ω*_ − 90° (Fig. 3f). These behaviors are consistent with the above prediction. Overall, Fig. 3d-f demonstrate that by changing *θ*_*ω*_ of the linearly polarized excitation light, spin-driven photocurrents with *θ*_*ω*_-invariant magnitude can be directionally controlled through 360° in the two-dimensional (2D) plane of the MM. This is clearly different from the helicity-dependent bidirectional photocurrents observed in the Co/(nonmagnet)/Pt magnetic heterostructures^34^.

**Fig. 4:**
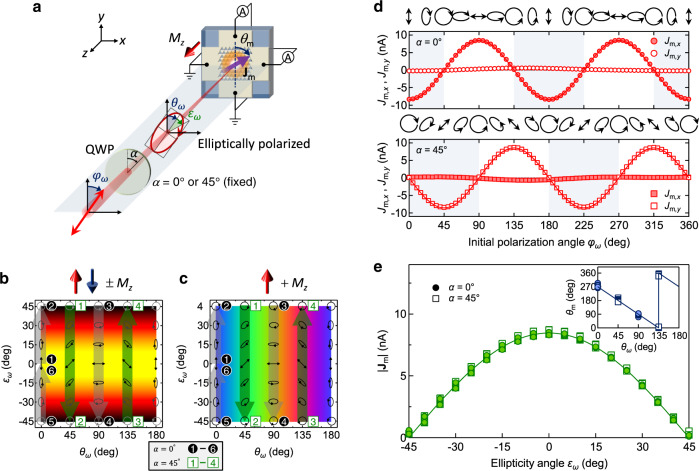
Simultaneous control of the magnitude and direction of spin-driven photocurrents. **a** Schematic showing the experimental setup for measuring zero-bias photocurrents along the *x* and *y* directions at normal incidence of continuously polarization-modulated 800-nm light. The polarization state of the excitation light is determined by the initial polarization angle *φ*_*ω*_ and rotation angle *α* of the optical axis of the quarter-wave plate (QWP). **b**, **c** 2D color maps of (**b**) ∣ **J**_m_∣ for ± *M*_*z*_ and (**c**) *θ*_m_ for + *M*_*z*_ when *φ*_*ω*_ crosses the vertical directions (marked by thick arrows). *ε*_*ω*_ can be continuously modulated with *θ*_*ω*_ = 0°/90° at *α* = 0° and *θ*_*ω*_ = 45°/135° at *α* = 45°, leading to the simultaneous control of ∣ **J**_m_∣ and *θ*_m_. **d** *φ*_*ω*_ dependence of *J*_m,*x*_ and *J*_m,*y*_ at *α* = 0° (upper panel) and *α* = 45° (lower panel). Shaded (nonshaded) areas correspond to *θ*_*ω*_ = 0° (*θ*_*ω*_ = 90°) at *α* = 0° and *θ*_*ω*_ = 45° (*θ*_*ω*_ = 135°) at *α* = 45°. **e**, $|{{{{{{{{{{{{\bf{\,J}}}}}}}}}}}}}_{{{{{{{{{{{{\rm{m}}}}}}}}}}}}} |=\sqrt{{(\,{J}_{{{{{{{{{{{{\rm{m}}}}}}}}}}}},x})}^{2}+{(\,{J}_{{{{{{{{{{{{\rm{m}}}}}}}}}}}},y})}^{2}}$ plotted as a function of *ε*_*ω*_. The line is fitted to $|{{{{{{{{{{{{\bf{\,J}}}}}}}}}}}}}_{{{{{{{{{{{{\rm{m}}}}}}}}}}}}}|\propto {{{{{{{{{\rm{cos}}}}}}}}}} 2{\varepsilon }_{\omega }$. The inset plots ${\theta }_{{{{{{{{{{{{\rm{m}}}}}}}}}}}}}={{{{{{{{{\rm{tan}}}}}}}}} }^{-1}\left(\,{J}_{{{{{{{{{{{{\rm{m}}}}}}}}}}}},x}/{J}_{{{{{{{{{{{{\rm{m}}}}}}}}}}}},y}\right)$ as a function of *θ*_*ω*_. The line is fitted to *θ*_m_ = − 2*θ*_*ω*_ − 90°.

Applying Eqs. (2) and (3), we further investigated whether a directional spin-driven photocurrent can be generated under an arbitrarily polarized light. For this purpose, we inserted a quarter-wave plate (QWP) after rotating the polarization angle *φ*_*ω*_ of the initially linearly polarized light, which modified both *θ*_*ω*_ and *ε*_*ω*_ of the excitation light (Fig. 4a). The polarization state of the excitation light is then determined by *φ*_*ω*_ and the rotation angle *α* of the QWP optical axis. In this setup, we can continuously modulate *ε*_*ω*_ with *θ*_*ω*_ = 0°/90° at *α* = 0° and *θ*_*ω*_ = 45°/135° at *α* = 45° (marked by the thick arrows in Fig. 4b, c, respectively), providing simultaneous control of ∣ **J**_m_∣ and *θ*_m_.

To confirm this prediction, we investigated the spin-driven photocurrents *J*_m,*x*_ and *J*_m,*y*_ as functions of *φ*_*ω*_. *J*_m,*x*_ (*J*_m,*y*_) shows $-{{{{{{{{{\rm{cos}}}}}}}}}}\, 2{\varphi }_{\omega }$ ($-{{{{{{{{{\rm{sin}}}}}}}}}}\, 2{\varphi }_{\omega }$) dependence on *φ*_*ω*_ whereas *J*_m,*y*_ (*J*_m,*x*_) is vanishingly small at *α* = 0° (*α* = 45°) (upper (lower) panel of Fig. 4d). When plotted as a function of *ε*_*ω*_, ∣ **J**_m_∣ exhibits the same behaviors at *α* = 0° and 45° (Fig. 4e); specifically, $|{{{{{{{{{{{{\bf{\,J}}}}}}}}}}}}}_{{{{{{{{{{{{\rm{m}}}}}}}}}}}}}|\propto {{{{{{{{{\rm{cos}}}}}}}}}} 2{\varepsilon }_{\omega }$. Therefore, the magnitude can be tuned from 100% to 0% by switching from linear (*ε*_*ω*_ = 0°) to circular (*ε*_*ω*_ = ± 45°) polarization. Meanwhile, *θ*_m_ can be independently controlled by *θ*_*ω*_ through the universal relation *θ*_m_ = − 2*θ*_*ω*_ − 90° at *α* = 0° and 45° (Fig. 4e, inset), as observed under linearly polarized light (Fig. 3f). Thus, the polarization information of an arbitrarily polarized light is converted into the magnitude and flow direction of spin-driven photocurrents.

## Discussion

Based on microscopic calculations, we briefly explain the mechanism of photo-driven rectified charge and spin currents. Considering the dependence on the intensity of the excitation light, the experimentally observed light-induced DC current originates from the second-order photoelectric field, as is the case for the MPGE. The present MPGE can be attributed to the magnetic injection current^15^

4 $${\beta }_{\mu ;\nu \lambda }^{{{{{{{{{{{{\rm{Inj}}}}}}}}}}}}}=\frac{-\pi {q}^{3}}{\gamma }\int \frac{d{{{{{{{{{{{\bf{k}}}}}}}}}}}}}{{\left(2\pi \right)}^{2}}\mathop{\sum}\limits_{a\ne b}\left({v}_{aa}^{\mu }-{v}_{bb}^{\mu }\right){g}_{ab}^{\nu \lambda }\frac{\gamma }{\pi }\frac{f({E}_{a})-f({E}_{b})}{{\gamma }^{2}+{(\hslash \omega -{E}_{b}+{E}_{a})}^{2}},$$

where *q* and *γ* are the elementary charge of carriers and the phenomenological scattering rate, respectively. *E*_*a*_(**k**) is the energy of one-particle, *f*(*x*) is the Fermi-Dirac distribution function, *v*^*μ*^ is the velocity operator, and *g*^*νλ*^ is the band-resolved quantum metric parametrized by the crystal momentum **k** and band indices *a* and *b*^35,36^. The injection current arises from resonant excitation of the electron-hole pairs and shows a diverging behavior in clean materials. As our MM is a conductor, intraband dynamics may also contribute to the (magneto-)photogalvanic response to linearly polarized light^36^. However, such intraband effects are expected to be negligible unless the frequency of light is as low as that of the THz waves^37^. Additionally, we ignore the Fermi-surface contribution because the frequency of the irradiating light is comparable to the bandwidth ( ~ 1 eV)^38^. The photogalvanic effect can be caused by the magnetic injection current only when the linearly polarized light is applied to systems where both space inversion and time-reversal symmetries are broken. These conditions are met by the MPGE discussed above.

In contrast, the spin photogalvanic effect (photo-driven rectified pure spin current) is formulated by calculating the spin current expectation value^36^. In the paramagnetic state denoted by the magnetic point group $3m1^{\prime}$, the dominant spin photogalvanic effect may occur due to the “spin” (linear) injection current denoted by

5 $${\tilde{\beta }}_{\alpha \mu ;\nu \lambda }^{{{{{{{{{{{{\rm{SpInj}}}}}}}}}}}}}=\frac{-\pi {q}^{2}}{\gamma }\int \frac{d{{{{{{{{{{{\bf{k}}}}}}}}}}}}}{{\left(2\pi \right)}^{2}}\mathop{\sum}\limits_{a\ne b}\left({j}_{aa}^{\alpha ;\mu }-{j}_{bb}^{\alpha ;\mu }\right){g}_{ab}^{\nu \lambda }\frac{\gamma }{\pi }\frac{f({E}_{a})-f({E}_{b})}{{\gamma }^{2}+{(\hslash \omega -{E}_{b}+{E}_{a})}^{2}},$$

which indicates the *α*-polarized spin current flow along the *μ*-direction under linearly polarized light. This formula is identical to that of the magnetic injection current (Eq. (4)) but with the velocity operator *v*^*μ*^ in Eq. (4) replaced with the spin current operator *j*^*α*;*μ*^. The spin current operator is naively defined as {*s*^*α*^, *v*^*μ*^}/2, where *s*^*α*^ is the spin operator. According to symmetry analysis, the allowed spin photogalvanic effect in the paramagnetic state is

6 $${\tilde{\beta }}_{zx;xx}=-{\tilde{\beta }}_{zx;yy}=-{\tilde{\beta }}_{zy;xy}=-{\tilde{\beta }}_{zy;yx},$$

for the *z*-polarized spin current and

7 $${\tilde{\beta }}_{xy;xx}=-{\tilde{\beta }}_{xy;yy}={\tilde{\beta }}_{yy;xy}={\tilde{\beta }}_{yy;yx}={\tilde{\beta }}_{yx;xx}=-{\tilde{\beta }}_{yx;yy}=-{\tilde{\beta }}_{xx;xy}=-{\tilde{\beta }}_{xx;yx},$$

for the spin current whose polarization is in the *xy* plane.

Notably, the *z*-polarized spin current exhibits the same polarization dependence as the MPGE. This coincidence can be intuitively understood by the following arguments. The large exchange splitting ( ~ 1 eV) in ferromagnetic cobalt causes nonequivalence of the spin-up and spin-down states. The photo-driven spin current is then converted into a strongly spin-polarized charge current, whereas the spin current carries no charge carriers in the paramagnetic state. Supporting this argument, both the *z*-polarized spin photocurrent and charge photocurrent follow the same polarization dependence originating from the same microscopic mechanism (namely, the injection mechanism). From these theoretical arguments on the experimental observations, the spin-polarized photocurrent is inferred to be controlled by the magnetic moment of the MM and the polarization state of the incident light.

The results of this proof-of-principle experiment newly demonstrate that nanoscale engineering of a standard spintronic thin film enables full manipulation of spin-driven photocurrents and can be applied to a broad range of ferro- and ferri-magnetic materials in general. The experimental results were entirely consistent with symmetry predictions, implying that our work can provide a guiding principle for the design and creation of artificially engineered opto-spintronic nanodevices and/or magnetic metasurfaces operating at room temperature. This study is complemented by ongoing successful explorations of novel magneto-optical and magnetoelectric functionalities in recently discovered 2D magnetic materials and heterostructures^39,40^. Widely tunable nanostructures and highly spin-polarized materials such as half metals may generate intense spin-polarized photocurrents. Combining the potential for ultrafast photocurrent generation^41^ with propagation through materials with long spin diffusion lengths (such as graphene)^42^, efficient spin-polarized currents could be transferred into a ferromagnet. This approach can potentially control the magnetization direction via a spin-transfer torque on sub-picosecond timescales^43^.

Our work can also guide the search for spin- and polarization-controllable THz devices and photovoltaic materials^15^. Recent studies have clarified the mechanisms of bulk photocurrent in magnetic systems and discussed applications for probing magnetic states^35,36^. However, magnetic domain formation is a serious problem in bulk magnets. For instance, the photocurrent developed no hysteresis in ferromagnetic topological insulators^6^. In the present paper, the spin-driven photocurrents due to large exchange splitting were demonstrated in a controllable way without magnetic domain formation and surpassed the photocurrents of nonmagnetic origin (see Supplementary Fig. 6). Thus, MMs are an ideal platform for future studies and applications of photocurrent generation on magnetic materials.

## Methods

### Preparation of magnetic metamaterial

To incorporate novel opto-spintronic functionality into a standard ferromagnet, we fabricated a MM consisting of periodic triangle-hole-arrayed nanostructures (antidot lattice) with threefold rotational symmetry (Fig. 1b). Onto a two-sided polished SiO_2_ substrate, we sequentially deposited a 5-nm-thick SiN layer, a 2-nm-thick Pt layer, a [Pt(0.9 nm)/Co(0.5 nm)]_5_ ferromagnetic multilayer, and a 2-nm-thick Pt layer. The deposition was performed by radio-frequency magnetron sputtering. Subsequently, triangular holes were created by electron beam lithography over an area of 250 × 250 *μ*m^2^, followed by Ar ion etching. The side lengths of each triangular hole and the period of the triangular lattice were 480 nm and 558 nm, respectively, sufficiently shorter than the wavelength of the excitation light. The fabricated MM was evaluated by an atomic force microscope (Fig. 1a, inset). The single triangular hole structure belongs to point group 3*m,* and its planar periodic array belongs to the *p*3*m*1 plane group with 3*m* point symmetry. At room temperature the Co/Pt ferromagnetic multilayer film exhibits out-of-plane magnetization that preserves the artificially built-in threefold rotational symmetry of the MM but breaks the time-reversal symmetry (the magnetic point group $3m^{\prime}$). Thus, our MM breaks both the space inversion and time-reversal symmetries, providing the necessary condition for observing the MPGE.

### Photocurrent measurements

To demonstrate the polarization-controlled tunable directional spin-driven photocurrents, the short-circuit photocurrents were measured across the unbiased MM (see Supplementary Fig. 1 for the device structure). The surrounding unstructured Co/Pt multilayer film was used as the electrodes. The excitation source was a Ti:sapphire ultrafast oscillator (Spectra-Physics, Mai Tai HP) operating at 690–1040 nm with a pulse width of 100 fs and a repetition rate of 80 MHz. The polarization state of the excitation light was controlled by a set of waveplates. A rotatable half-wave plate controlled the polarization angle *θ*_*ω*_ of the incident light and an optional QWP modulated the ellipticity angle *ε*_*ω*_ simultaneously with *θ*_*ω*_ (Fig. 4a). The MM was mounted in an electromagnet and its out-of-plane magnetization *M*_*z*_ was controlled by applying out-of-plane magnetic fields. The laser beam was delivered to the MM at normal incidence using an optical microscope. The spot delivered to the MM ( ~ 250 μm) was comparable in size to the nanostructured region. The laser power irradiated on the MM was typically ~ 100 mW, corresponding to a peak intensity of ~ 200 Wcm^−2^. The laser beam passed through the MM to a charge-coupled device camera that monitored its position on the MM. The laser spot was carefully positioned at the center of the MM to exclude laser heating-induced thermoelectric currents such as the Seebeck effect^5^ and the anomalous Nernst effect^44^. The intensity of the laser beam was modulated at *f* ~ 1 kHz by an optical chopper. The short-circuit photocurrents were separately measured along the *x* and *y* directions by a two-phase lock-in amplifier (Stanford Research Systems, SR865) with the reference signal from the optical chopper. Thus, the photocurrent signals were evaluated as a time-averaged discharge current from the MM. The magnitude ∣ **J**_m_∣ and flow direction *θ*_m_ of the spin-driven photocurrents were calculated as $|{{{{{{{{{{{{\bf{\,J}}}}}}}}}}}}}_{{{{{{{{{{{{\rm{m}}}}}}}}}}}}} |=\sqrt{{(\,{J}_{{{{{{{{{{{{\rm{m}}}}}}}}}}}},y})}^{2}+{\,(\,{J}_{{{{{{{{{{{{\rm{m}}}}}}}}}}}},y})}^{2}}$ and ${\theta }_{{{{{{{{{{{{\rm{m}}}}}}}}}}}}}={\tan }^{-1}(\,{J}_{{{{{{{{{{{{\rm{m}}}}}}}}}}}},x}/{J}_{{{{{{{{{{{{\rm{m}}}}}}}}}}}},y})$, respectively.

The dynamical response of the photocurrents was measured with a digitizing storage oscilloscope (LeCroy, WaveRunner8104; bandwidth of 1 GHz) through a wide-band preamplifier (Stanford Research Systems, SR445A; bandwidth of 350 MHz). During the measurements, the MM was illuminated by a Ti:sapphire regenerative amplifier system (Spectra-Physics, Hurricane) operating at 800 nm with a pulse width of ~ 130 fs and a repetition rate of 1 kHz. The laser power irradiated on the MM was typically ~ 0.1 mW ( ~ 0.1 μJ/pulse). The time trace of the photocurrent was averaged 5000 times. The response was broadened by the response time of the preamplifier and was followed by oscillations (Fig. 2c) due to impedance mismatch in the circuit. All measurements were performed at room temperature in air.

### Faraday rotation measurements

The magnetization curves of the unstructured Co/Pt multilayer film and MM were evaluated using Faraday rotation measurements under the 800 nm laser light used for the photocurrent measurements (see Fig. 2e and Supplementary Fig. 2). Due to the Faraday effect, the plane of the linearly polarized light rotates after passing through the magnetic materials. The Faraday rotation angle *θ*_F_, measured using a conventional balanced detection technique, is proportional to *M*_*z*_.

### Linear optical measurements

The linear transmittance (*T*) and reflectivity (*R*) spectra of the unstructured Co/Pt multilayer film and MM were measured over the 250–2500 nm range using a commercial spectrometer (JASCO, MSV-5200). The absorption (*A*) spectra were calculated as *A* = 1 − *T* − *R* (see Supplementary Fig. 5).

### Symmetry analysis of the magneto-photogalvanic effect

Under the magnetic point group of $3m^{\prime}$ for the MM with a spontaneous magnetization parallel to the threefold rotational axis, the MPGE has four independent nonzero tensor components^30^: ${\beta }_{xxx}^{{{{{{{{{{{{\rm{m}}}}}}}}}}}}}=-{\beta }_{xyy}^{{{{{{{{{{{{\rm{m}}}}}}}}}}}}}=-{\beta }_{yxy}^{{{{{{{{{{{{\rm{m}}}}}}}}}}}}}=-{\beta }_{yyx}^{{{{{{{{{{{{\rm{m}}}}}}}}}}}}}\equiv {\beta }^{{{{{{{{{{{{\rm{m}}}}}}}}}}}}}$, ${\beta }_{xyz}^{{{{{{{{{{{{\rm{m}}}}}}}}}}}}}=-{\beta }_{yxz}^{{{{{{{{{{{{\rm{m}}}}}}}}}}}}}$, ${\beta }_{xzy}^{{{{{{{{{{{{\rm{m}}}}}}}}}}}}}=-{\beta }_{yzx}^{{{{{{{{{{{{\rm{m}}}}}}}}}}}}}$, ${\beta }_{zxy}^{{{{{{{{{{{{\rm{m}}}}}}}}}}}}}=-{\beta }_{zyx}^{{{{{{{{{{{{\rm{m}}}}}}}}}}}}}$. When a laser light ${{{{{{{{{{{\bf{E}}}}}}}}}}}}(\omega )={{{{{{{{{{{{\bf{E}}}}}}}}}}}}}_{0}{e}^{-i\left(kz-\omega t\right)}$ propagating along the − *z* direction is irradiated on the MM at normal incidence (Fig. 1a), only *β*^m^ contributes to the spin-driven photocurrents in the 2D plane of the MM. Under arbitrarily polarized light irradiation with ${{{{{{{{{{\bf{E}}}}}}}}}}}_{0}=\left({E}_{x},{E}_{y},{E}_{z}\right)=\left({{{{{{\rm{sin}}}}}}}\, {\theta }_{\omega }{{{{{{\rm{cos}}}}}}}\, {\varepsilon }_{\omega }-i\,{{{{{{\rm{cos}}}}}}}\, {\theta }_{\omega }{{{{{{\rm{sin}}}}}}}\, {\varepsilon }_{\omega },{{{{{{\rm{cos}}}}}}}\, {\theta }_{\omega } {{{{{{\rm{cos}}}}}}}\, {\varepsilon }_{\omega }+i\,{{{{{{\rm{sin}}}}}}}\, {\theta }_{\omega } {{{{{{\rm{sin}}}}}}}\, {\varepsilon }_{\omega },0\right)$, spin-driven photocurrents are generated along the *x* and *y* directions as

8 $${J}_{{{{{{{{{{{{\rm{m}}}}}}}}}}}},x}={\beta }^{{{{{{{{{{{{\rm{m}}}}}}}}}}}}}\left({E}_{x}{E}_{x}^{*}-{E}_{y}{E}_{y}^{*}\right)=-{\beta }^{{{{{{{{{{{{\rm{m}}}}}}}}}}}}}{{{{{{{{\rm{cos}}}}}}}}}\, 2{\theta }_{\omega }{{{{{{{{\rm{cos}}}}}}}}}\, 2{\varepsilon }_{\omega },$$

9 $${J}_{{{{{{{{{{{{\rm{m}}}}}}}}}}}},y}=-{\beta }^{{{{{{{{{{{{\rm{m}}}}}}}}}}}}}\left({E}_{x}{E}_{y}^{*}+{E}_{y}{E}_{x}^{*}\right)=-{\beta }^{{{{{{{{{{{{\rm{m}}}}}}}}}}}}}{{{{{{{{\rm{sin}}}}}}}}}\; 2{\theta }_{\omega }{{{{{{{{\rm{cos}}}}}}}}}\; 2{\varepsilon }_{\omega }.$$

From these expressions, we get

10 $$|{{{{{{{{{{{{\bf{\,J}}}}}}}}}}}}}_{{{{{{{{{{{{\rm{m}}}}}}}}}}}}} |=\sqrt{{\left(\,{J}_{{{{{{{{{{{{\rm{m}}}}}}}}}}}},x}\right)}^{2}+{\left(\,{J}_{{{{{{{{{{{{\rm{m}}}}}}}}}}}},y}\right)}^{2}}=\left|{\beta }^{{{{{{{{{{{{\rm{m}}}}}}}}}}}}}\right|{{{{{{{{\rm{cos}}}}}}}}}\, 2{\varepsilon }_{\omega }\qquad (-4{5}^{\circ }\le {\varepsilon }_{\omega }\le 4{5}^{\circ }),$$

and

11 $${\theta }_{{{{{{{{{{{{\rm{m}}}}}}}}}}}}}=\left\{\begin{array}{ll}-2{\theta }_{\omega }\mp 9{0}^{\circ }\qquad \quad &({\beta }^{{{{{{{{{{{{\rm{m}}}}}}}}}}}}} \, > \, 0)\\ -2{\theta }_{\omega }\pm 9{0}^{\circ }\qquad \quad &({\beta }^{{{{{{{{{{{{\rm{m}}}}}}}}}}}}} \, < \, 0)\end{array}\right.,$$

where ∓ and ± in Eq. (11) depends on the magnetization direction ± *M*_*z*_. Equations (10) and (11) imply that ∣ **J**_m_∣ and *θ*_m_ of the spin-driven photocurrents can be independently controlled by *ε*_*ω*_ and *θ*_*ω*_ of the excitation light, respectively, and that *θ*_m_ flips when *M*_*z*_ reverses. Thus, the polarization information of an arbitrarily polarized light can be converted into the magnitude and flow direction of the spin-driven photocurrents. These relations are purely determined by the artificially built-in symmetry of the MM and are not restricted to specific material properties or excitation wavelength^31^, thus allowing broad-band operation (see Supplementary Fig. 6). The conventional (nonmagnetic) photogalvanic effect is discussed in Supplementary Figs. 3, 4, 6 and Supplementary Discussion.

## Supplementary information

Supplementary Information

## Data Availability

The data that support the findings of this study are available from the corresponding author upon reasonable request.
