# Supplementary material for: Polarization-controlled tunable directional spin-driven photocurrents in a magnetic metamaterial with threefold rotational symmetry
Source: Nat Commun. 2022 Nov 7;13:6708. doi: 10.1038/s41467-022-34374-7 (PMC9640558; doi:10.1038/s41467-022-34374-7)
Supplement: Supplementary file 1 — Supplementary Information [file 41467_2022_34374_MOESM1_ESM.pdf]

Supplementary Information for

Polarization-controlled tunable directional spin-driven photocurrents in a magnetic metamaterial with threefold rotational symmetry

Masakazu Matsubara[1,2*], Takatsugu Kobayashi[1], Hikaru Watanabe[3], Youichi Yanase[4,5], Satoshi Iwata[6], Takeshi Kato[6]

[1] *Department of Physics, Tohoku University, Sendai 980-8578, Japan*

[2] *Center for Science and Innovation in Spintronics, Tohoku University, Sendai 980-8577, Japan*

[3] *Research Center for Advanced Science and Technology, University of Tokyo, Tokyo 153-8904, Japan*

[4] *Department of Physics, Graduate School of Science, Kyoto University, Kyoto 606-8502, Japan*

[5] *Institute for Molecular Science, Okazaki 444-8585, Japan*

[6] *Institute of Materials and Systems for Sustainability, Nagoya University, Furo-cho, Chikusa-ku, Nagoya 464-8603, Japan*

[*]E-mail: m-matsubara@tohoku.ac.jp

Supplementary Figures

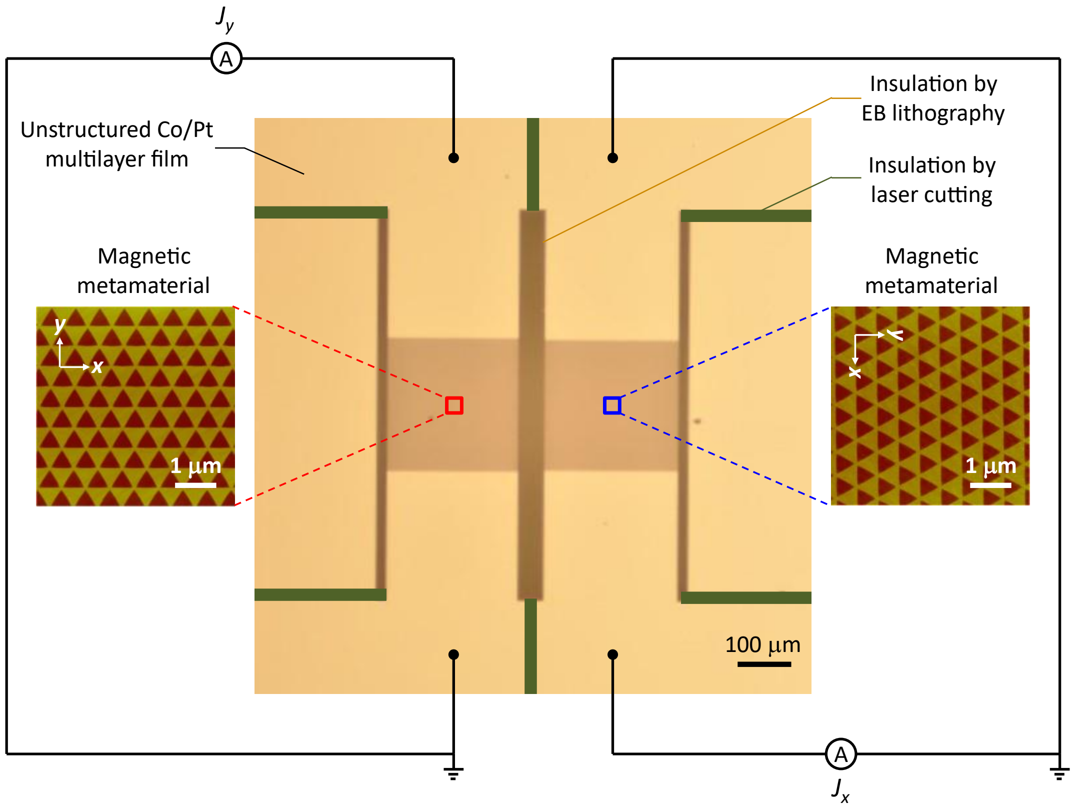

Supplementary Figure 1 | Magnetic metamaterial (MM) device for polarization-controlled tunable directional photocurrent generation. Optical microscope image of the central region of the MM device and a representation of its electrical connections. The periodic triangle-hole-arrayed nanostructures (antidot lattice) with threefold rotational symmetry were created by electron beam (EB) lithography (area = 250 × 250 μm^2) in the unstructured Co/Pt ferromagnetic multilayer film on a SiO_2 substrate. The side lengths of each triangular hole and the period of the triangular lattice are 480 nm and 558 nm, respectively, which are evaluated by an atomic force microscope (insets). Two MMs were arranged in a 90° tilted configuration for photocurrent measurements along the y (left inset) and x (right inset) directions. The surrounding unstructured Co/Pt multilayer film was used as the electrodes. The MMs for the J_y and J_x measurements were electrically separated by a central insulation line created by EB lithography and additional insulation lines formed by laser cutting (schematically drawn by green lines). The laser beam was delivered to each MM at normal incidence using an optical microscope; the resulting spots were sized ~250 μm, comparable to the size of the nanostructured region. The short-circuit photocurrents were separately measured along the x and y directions of each MM.

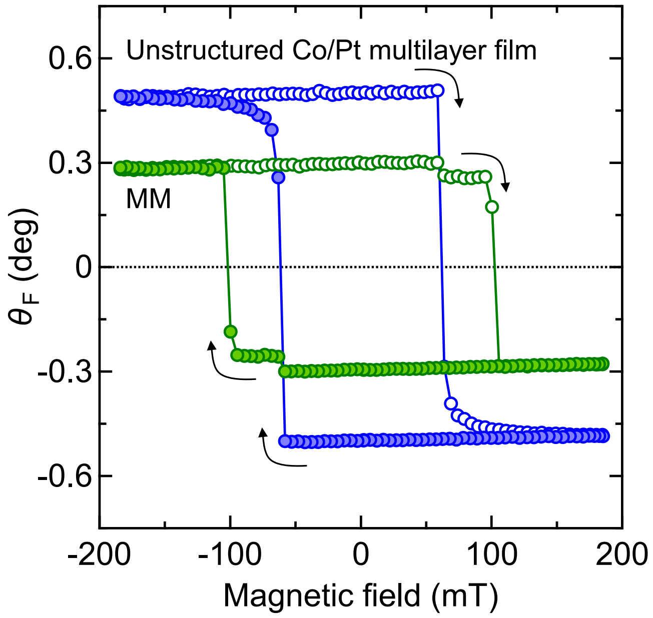

Supplementary Figure 2 | Modulation of the coercive field by nanofabrication. Out-of-plane magnetic-field dependence of Faraday rotation angle θ_F of the unstructured Co/Pt multilayer film and MM at room temperature. The coercive field H_c of the MM (H_c ~100 mT) exceeds that of the unstructured Co/Pt multilayer film (H_c ~60 mT) because the smooth movement of the magnetic domain walls is disturbed by the nanostructures in the MM. During the MM measurements, the incident laser beam slightly hits the surrounding unstructured Co/Pt multilayer film. This contribution from the unstructured Co/Pt multilayer film causes the small step features in the θ_F plot around ±60 mT. This feature is absent in the photocurrent measurements in Fig. 2d of the main text because the unstructured Co/Pt multilayer film has a centrosymmetric crystal structure and the photocurrents are not contributed by the magneto-photogalvanic effect, which requires the breaking of space inversion symmetry.

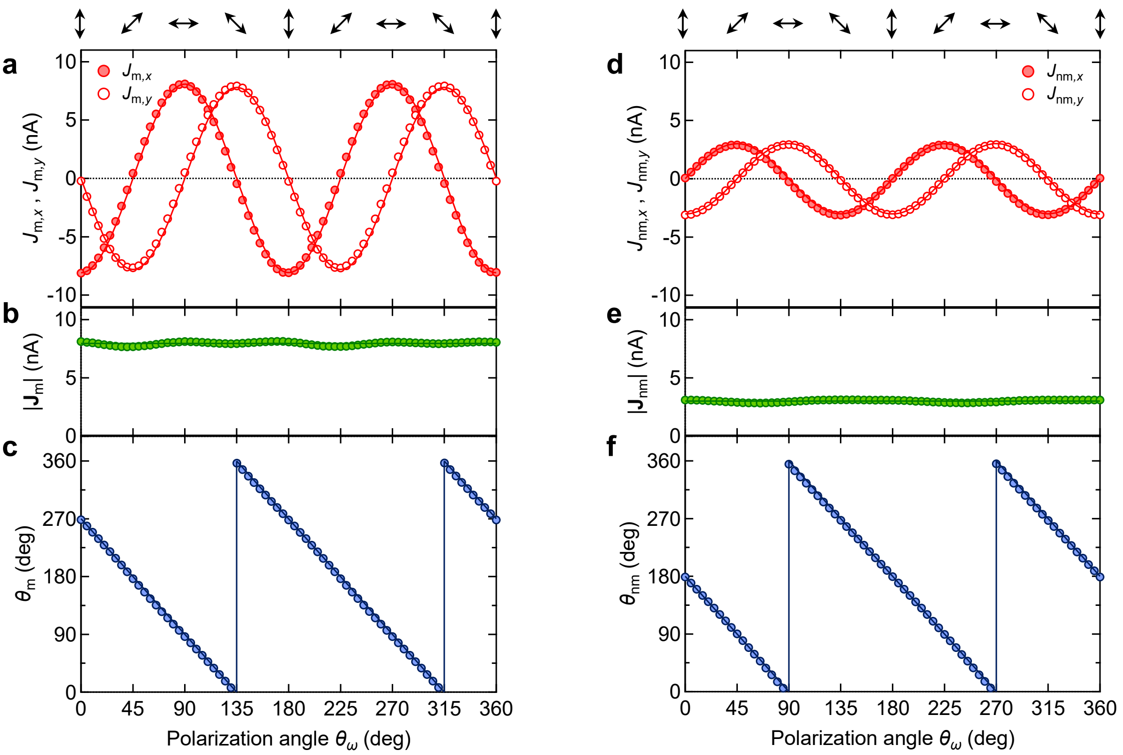

Supplementary Figure 3 | Full-directional control of spin-driven and nonmagnetic photocurrents by linearly polarized light. Zero-bias photocurrents were measured along the x and y directions at normal incidence of continuously rotated linearly polarized 800-nm light. The experimental configuration is that of Fig. 3a in the main text. Plotted are the θ_ω dependences of (**a-c**) the spin-driven photocurrents $\mathbf{J}_\mathrm{m}$ generated via the magneto-photogalvanic effect and (**d-f**) the nonmagnetic photocurrents $\mathbf{J}_\mathrm{nm}$ generated via the conventional photogalvanic effect. The results in (**a-c**) are reproduced from Fig. 3d-f in the main text for direct comparison between $\mathbf{J}_\mathrm{m}$ and $\mathbf{J}_\mathrm{nm}$. (**d**) θ_ω dependence of $J_{\mathrm{nm},x}$ and $J_{\mathrm{nm},y}$, calculated as $J_{\mathrm{nm},x} = [J_x(+M_z) + J_x(-M_z)]/2$ and $J_{\mathrm{nm},y} = [J_y(+M_z) + J_y(-M_z)]/2$, respectively. The lines are fitted to $J_{\mathrm{nm},x} \propto \sin 2\theta_\omega$ and $J_{\mathrm{nm},y} \propto -\cos 2\theta_\omega$. (**e**) $|\mathbf{J}_\mathrm{nm}|$ calculated as $|\mathbf{J}_\mathrm{nm}| = \sqrt{(J_{\mathrm{nm},x})^2 + (J_{\mathrm{nm},y})^2}$ and $|\mathbf{J}_\mathrm{nm}| = \text{const.}$ (**f**) θ_nm calculated as $\theta_\mathrm{nm} = \tan^{-1}(J_{\mathrm{nm},x}/J_{\mathrm{nm},y})$. The line is fitted to $\theta_\mathrm{nm} = -2\theta_\omega + 180^\circ$. Laser power is 100 mW in all cases.

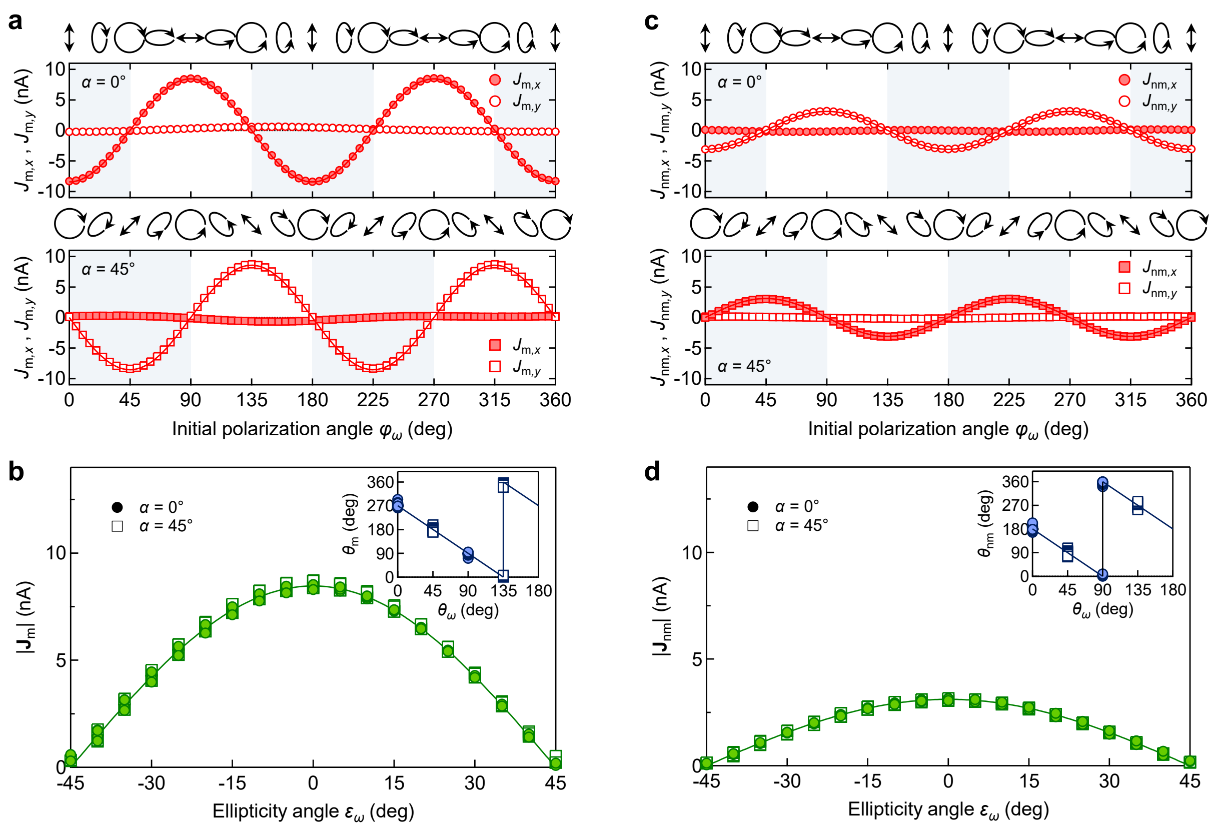

Supplementary Figure 4 | Simultaneous control of the magnitude and direction of spin-driven and nonmagnetic photocurrents. Zero-bias photocurrents were measured along the x and y directions at normal incidence of continuously polarization-modulated 800-nm light. The experimental configuration is that of Fig. 4a in the main text. The polarization state of the excitation light is determined from the initial polarization angle φ_ω and rotation angle α of the optical axis of the quarter-wave plate. Plotted are the φ_ω dependences of (**a**, **b**) the spin-driven photocurrents $\mathbf{J}_\mathrm{m}$ generated via the magneto-photogalvanic effect and (**c**, **d**) the nonmagnetic photocurrents $\mathbf{J}_\mathrm{nm}$ generated via the conventional photogalvanic effect. The results in (**a**, **b**) are reproduced from Fig. 4d and e in the main text for direct comparison between $\mathbf{J}_\mathrm{m}$ and $\mathbf{J}_\mathrm{nm}$. (**c**) φ_ω dependence of $J_{\mathrm{nm},x}$ and $J_{\mathrm{nm},y}$ at $\alpha = 0^\circ$ (upper panel) and $\alpha = 45^\circ$ (lower panel). Shaded (nonshaded) areas correspond to $\theta_\omega = 0^\circ$ ($\theta_\omega = 90^\circ$) at $\alpha = 0^\circ$ and $\theta_\omega = 45^\circ$ ($\theta_\omega = 135^\circ$) at $\alpha = 45^\circ$. (**d**) Plot of $|\mathbf{J}_\mathrm{nm}| = \sqrt{(J_{\mathrm{nm},x})^2 + (J_{\mathrm{nm},y})^2}$ versus ε_ω. The line is fitted to $|\mathbf{J}_\mathrm{nm}| \propto \cos 2\varepsilon_\omega$. Inset: Plot of $\theta_\mathrm{nm} = \tan^{-1}(J_{\mathrm{nm},x}/J_{\mathrm{nm},y})$ versus θ_ω. The line is fitted to $\theta_\mathrm{nm} = -2\theta_\omega + 180^\circ$. Laser power is 100 mW in all cases.

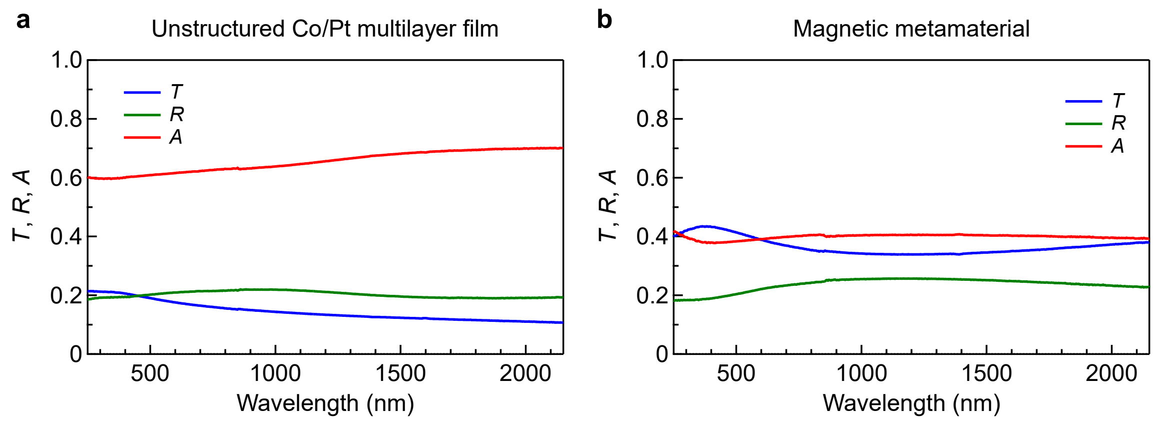

Supplementary Figure 5 | Linear optical properties. Linear transmittance (T) and reflectivity (R) spectra of (**a**) the unstructured Co/Pt multilayer film and (**b**) the magnetic metamaterial (MM) under linearly polarized light at room temperature. The absorption (A) spectra were calculated as $A = 1 - T - R$. Owing to the threefold rotational symmetry of the MM, the spectral profile is independent of the polarization state of the light.

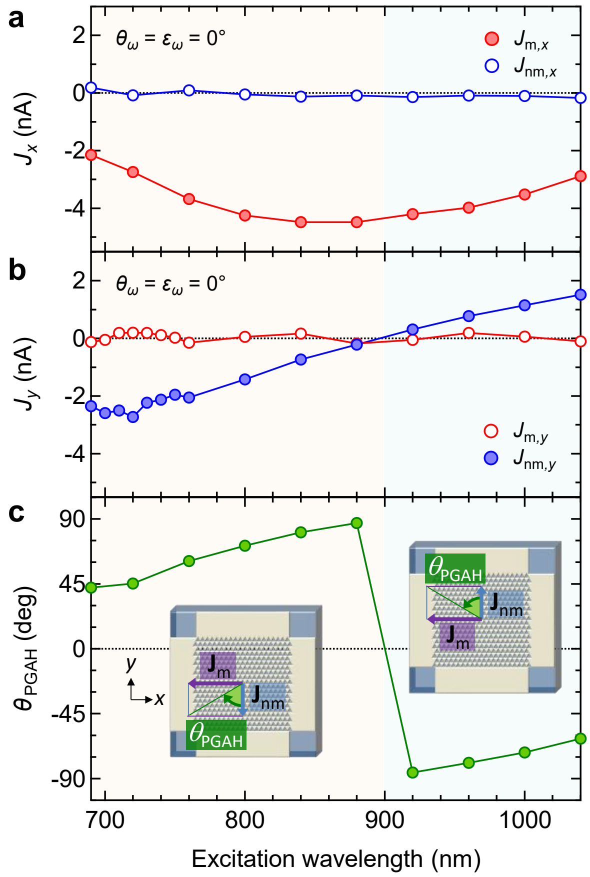

Supplementary Figure 6 | Excitation-wavelength dependence of spin-driven and nonmagnetic photocurrents. (**a**, **b**) Zero-bias photocurrents along the x and y directions of the MM at normal incidence of linearly y-polarized light at room temperature ($\theta_\omega = \varepsilon_\omega = 0^\circ$; excitation wavelength = 690–1040 nm). The spin-driven photocurrents $J_{\mathrm{m},x}$ and $J_{\mathrm{m},y}$ were calculated as $J_{\mathrm{m},x} = [J_x(+M_z) - J_x(-M_z)]/2$ and $J_{\mathrm{m},y} = [J_y(+M_z) - J_y(-M_z)]/2$, respectively, whereas the nonmagnetic photocurrents $J_{\mathrm{nm},x}$ and $J_{\mathrm{nm},y}$ were calculated as $J_{\mathrm{nm},x} = [J_x(+M_z) + J_x(-M_z)]/2$ and $J_{\mathrm{nm},y} = [J_y(+M_z) + J_y(-M_z)]/2$, respectively. In this experimental configuration, $\mathbf{J}_\mathrm{m}$ ($\mathbf{J}_\mathrm{nm}$) appeared only along the x (y) direction over the whole wavelength region, as expected from symmetry predictions and actually observed. These relations are purely determined by the artificially built-in symmetry of the MM and are restricted neither to the specific material properties nor the excitation wavelength. (**c**) Photogalvanic anomalous Hall angle calculated as $\theta_{\mathrm{PGAH}} = \tan^{-1}(J_{\mathrm{m},x}/J_{\mathrm{nm},y})$, versus excitation wavelength. Interestingly, θ_{PGAH} exceeded 45° over most of the wavelength range and reached $\pm 90^\circ$ around 900 nm, indicating that the spin-driven photocurrent $\mathbf{J}_\mathrm{m}$ generated via the magneto-photogalvanic effect became larger than the nonmagnetic photocurrent $\mathbf{J}_\mathrm{nm}$ generated via the conventional photogalvanic effect. The origin and wavelength dependence of this behavior must be theoretically elucidated in future work. The laser power at all wavelengths was 50 mW.

Supplementary Discussion

Symmetry analysis of the conventional (nonmagnetic) photogalvanic effect.

The MM has a $3m$ point group that admits four independent nonzero tensor components for the conventional (nonmagnetic) photogalvanic effect: $\beta_{xxy}^{\mathrm{nm}} = \beta_{xyx}^{\mathrm{nm}} = \beta_{yxx}^{\mathrm{nm}} = -\beta_{yyy}^{\mathrm{nm}} \equiv -\beta^{\mathrm{nm}}, \beta_{xxz}^{\mathrm{nm}}, \beta_{xzx}^{\mathrm{nm}}, \beta_{yyz}^{\mathrm{nm}}, \beta_{yzy}^{\mathrm{nm}}, \beta_{zxx}^{\mathrm{nm}} = \beta_{zyy}^{\mathrm{nm}}, \beta_{zzz}^{\mathrm{nm}}$. When a laser light $\mathbf{E}(\omega) = \mathbf{E}_0 e^{-i(kz-\omega t)}$ propagating along the $-z$ direction is irradiated on the MM at normal incidence (Fig. 1a in the main text), only β^{nm} contributes to the nonmagnetic photocurrents in the 2D plane of the MM. Under arbitrarily polarized light irradiation with $\mathbf{E}_0 = (E_x, E_y, E_z) = (\sin\theta_\omega \cos\varepsilon_\omega - i\cos\theta_\omega \sin\varepsilon_\omega, \cos\theta_\omega \cos\varepsilon_\omega + i\sin\theta_\omega \sin\varepsilon_\omega, 0)$, the nonmagnetic photocurrents are generated along the x and y directions as

$$J_{\mathrm{nm},x} = -\beta^{\mathrm{nm}}(E_x E_y^* + E_y E_x^*) = -\beta^{\mathrm{nm}} \sin 2\theta_\omega \cos 2\varepsilon_\omega\,, \qquad (S1)$$

$$J_{\mathrm{nm},y} = -\beta^{\mathrm{nm}}(E_x E_x^* - E_y E_y^*) = \beta^{\mathrm{nm}} \cos 2\theta_\omega \cos 2\varepsilon_\omega\,. \qquad (S2)$$

From these expressions, we get

$$|\mathbf{J}_{\mathrm{nm}}| = \sqrt{(J_{\mathrm{nm},x})^2 + (J_{\mathrm{nm},y})^2} = |\beta^{\mathrm{nm}}| \cos 2\varepsilon_\omega \quad (-45^\circ \le \varepsilon_\omega \le 45^\circ), \qquad (S3)$$

and

$$\theta_{\mathrm{nm}} = \tan^{-1}\left(\frac{J_{\mathrm{nm},x}}{J_{\mathrm{nm},y}}\right) = \begin{cases} -2\theta_\omega & (\beta^{\mathrm{nm}} > 0) \\ -2\theta_\omega + 180^\circ & (\beta^{\mathrm{nm}} < 0) \end{cases}. \qquad (S4)$$

Equations (S3) and (S4) indicate that $|\mathbf{J}_{\mathrm{nm}}|$ and θ_{nm} of the nonmagnetic photocurrents can be independently controlled by ε_ω and θ_ω of the excitation light, respectively. The spin-driven photocurrents generated via the magneto-photogalvanic effect can be controlled similarly, but the above expressions do not depend on the magnetization direction $\pm M_z$. Instead, they are purely determined by the artificially built-in symmetry of the MM and are not restricted to specific material properties or excitation wavelength (see Supplementary Fig. 6).
